# biMM: efficient estimation of genetic variances and covariances for cohorts with high-dimensional phenotype measurements

**DOI:** 10.1093/bioinformatics/btx166

**Published:** 2017-03-28

**Authors:** Matti Pirinen, Christian Benner, Pekka Marttinen, Marjo-Riitta Järvelin, Manuel A Rivas, Samuli Ripatti

**Affiliations:** 1 Institute for Molecular Medicine Finland (FIMM), University of Helsinki, Helsinki, Finland; 2 Helsinki Institute for Information Technology HIIT and Department of Mathematics and Statistics, University of Helsinki, Helsinki, Finland; 3 Department of Public Health, University of Helsinki, Helsinki, Finland; 4 Helsinki Institute for Information Technology HIIT and Department of Computer Science, Aalto University, Espoo, Finland; 5 Biocenter Oulu, University of Oulu, Oulu, Finland; 6 Department of Epidemiology and Biostatistics, MRC-PHE Centre for Environment and Health, School of Public Health, Imperial College London, London, UK; 7 Center for Life Course and Systems Epidemiology, Faculty of Medicine, University of Oulu, Oulu, Finland; 8 Unit of Primary Care, Oulu University Hospital, Oulu, Finland; 9 Department of Biomedical Data Science, Stanford University, Stanford, CA, USA

## Abstract

**Summary:**

Genetic research utilizes a decomposition of trait variances and covariances into genetic and environmental parts. Our software package biMM is a computationally efficient implementation of a bivariate linear mixed model for settings where hundreds of traits have been measured on partially overlapping sets of individuals.

**Availability and Implementation:**

Implementation in R freely available at www.iki.fi/mpirinen.

**Supplementary information:**

Supplementary data are available at *Bioinformatics* online.

## 1 Introduction

Decomposing phenotypic variance and covariance into genetic and environmental parts is important for designing genetic studies and understanding relationships between traits and diseases. The two main approaches are linear mixed model (LMM) implementations, such as GCTA (Yang *et al.*, 2011), GEMMA (Zhou and Stephens, 2014) or BOLT-REML (Loh *et al.*, 2015) and LD-score regression, implemented in LDSC (Bulik-Sullivan *et al.*, 2015). LMM requires access to the individual-level genotype-phenotype data whereas LDSC only needs output from a genome-wide association study (GWAS) and variant correlations from a reference database, but consequently may be less precise than LMM (Bulik-Sullivan *et al.*, 2015).

We consider settings where individual-level data are available, and hence use LMM. The bivariate LMM for *n* individuals is Y=G+ε, where $Y={\left(Y_{1}^{T},Y_{2}^{T}\right)}^{T}$ is 2*n*-vector of mean-centered phenotype values from which the covariates, such as age, sex and principal components of population structure have been regressed out, $G∼N(0,\Sigma_{G})$ is 2*n*-vector of genetic random effects and $\varepsilon ∼N(0,\Sigma_{\varepsilon })$ is 2*n*-vector of environmental random effects. The (2n)×(2n) covariance structures are parameterized by six scalars: genetic variances $V_{G1}$ and $V_{G2}$, genetic covariance $V_{G12}$, environmental variances $V_{\varepsilon 1}$ and $V_{\varepsilon 2}$ and environmental covariance $V_{\varepsilon 12}$ as

$$\Sigma_{G}=\left[\begin{array}{cc} V_{G1}R & V_{G12}R\\ V_{G12}R & V_{G2}R \end{array}\right]\;\text{and}\;\Sigma_{\varepsilon }=\left[\begin{array}{cc} V_{\varepsilon 1}I & V_{\varepsilon 12}I\\ V_{\varepsilon 12}I & V_{\varepsilon 2}I \end{array}\right]$$

expressed as *n *×* n* block matrices. I is the identity matrix and the element *i*, *j* of the genetic relationship matrix (GRM) R is

$$R_{ij}=\frac{1}{K}\sum_{k=1}^{K}\left(g_{ik}-2\hat{f_{k}}\right)\left(g_{jk}-2\hat{f_{k}}\right){\left(2\hat{f_{k}}\left(1-\hat{f_{k}}\right)\right)}^{\alpha },$$

where *g_ik_* is the genotype of individual *i* at variant *k*, coded as 0, 1 or 2 copies of the minor allele and $\hat{f_{k}}$ is the minor allele frequency (MAF). We use the standard scaling of allelic effects determined by α=−1.

From this model, an estimate of *V_Gt_* approximates additive genetic variance of each trait (*t* = 1, 2) explained by the variants included in the calculation of R and is often used as a lower bound for the (narrow-sense) heritability (detailed assumptions in Yang *et al.*, 2015). An estimate of the genetic correlation $\rho_{G}=V_{G12}/\sqrt{V_{G1}V_{G2}}$ measures (average) correlation of the allelic effects of the variants on the two traits. Similarly, we can estimate $\rho_{\varepsilon }=V_{\varepsilon 12}/\sqrt{V_{\varepsilon 1}V_{\varepsilon 2}}$, the correlation in the environmental components between the traits.

The existing bivariate LMM implementations have not been designed for a case where hundreds of traits have been measured on 10 000s of individuals. Our software package biMM combines a fast likelihood computation (similar in speed to GEMMA) with an algorithm that optimizes the sample overlap between consecutive pairs of traits analyzed and therefore efficiently reuses the computationally expensive matrix decompositions. biMM allows user to control how much missing data are tolerated for a single analysis and automatically executes both phenotype imputation and matrix decompositions required to achieve that tolerance.

## 2 Materials and methods

### 2.1 Reusing eigendecomposition

Once an eigendecomposition of R is available, our biMM algorithm drops the time complexity from cubic to quadratic for a trait pair and from cubic to linear for a single evaluation of the likelihood function (Supplementary Information). Similar time complexity is achieved by GEMMA, and efficient algorithms for multivariate LMM have recently been considered also by Furlotte and Eskin (2015) and Casale *et al.* (2015). Our central observation is that a complete sample overlap between two trait pairs means that the same eigendecomposition can be used for both pairs. To fully utilize this observation, we need to keep the eigendecomposition in random access memory (RAM) across the trait pairs and we need to optimize the order of the trait pairs. To our knowledge, neither of these functionalities is available in existing software.

### 2.2 Ordering pairs, imputing and dropping values

We order the trait pairs in such a way that the consecutive pairs have a large sample overlap. biMM further allows imputing at most *t_i_* missing values and/or dropping at most *t_d_* non-missing values for a trait pair to make it match the available eigendecomposition (Supplementary Information). Only when this is not possible for any remaining pair does biMM a new eigendecomposition. Algorithmically, given user-specified *t_i_* and *t_d_*, biMM finds an ordering that results in a small number of total eigendecompositions. This is an instance of the shortest Hamiltonian path problem that we tackle by a greedy heuristic (Supplementary Information).

### 2.3 Example analyses

We consider data from the Northern Finland Birth Cohort 1966 (NFBC1966) (Rantakallio *et al.*, 1969) with 16 traits having sample sizes between 4736 and 5025 individuals (Supplementary Table S1) and preprocessed by Tukiainen *et al.* (2014). We analyzed all 120 pairs of traits using both the complete ($t_{i}=t_{d}=0$) and an approximate versions ($t_{i}=t_{d}=200$) of biMM and compared with GCTA 1.25.3, GEMMA 0.94.1 and BOLT-REML 2.2 with their default parameters. biMM ran in R-3.3.1 with Intel Math Kernel Library.

To assess scaling to larger datasets, we consider 20 000 individuals simulated by HapGen2 (Su *et al.*, 2011) using chromosome 2 of the CEU panel from HapMap3 (International HapMap3 consortium., 2010) with phenotypes generated to have heritabilities between 0.2 and 0.8.

In all examples we used a desktop computer with an Intel Quad-Core i7-3770 CPU @ 3.40 GHz and 16 Gb of RAM.

## 3 Results

**Fig. 1:**
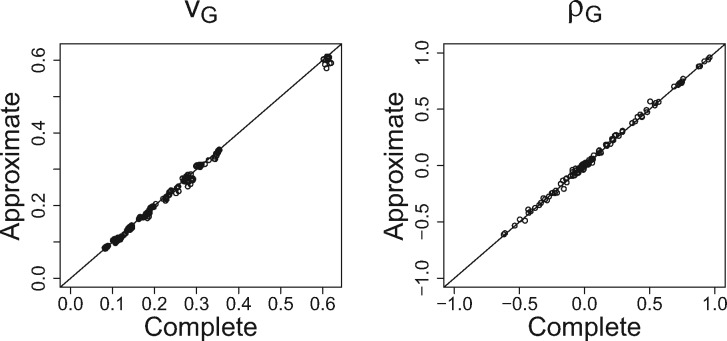
Comparing estimates for heritability (*V_G_*) and genetic correlation (*ρ_G_*) between an approximate ($t_{i}=t_{d}=200$) and complete ($t_{i}=t_{d}=0$) versions of biMM over 120 pairs of traits

Figure 1 shows that the complete and approximate versions of biMM are very similar across the 120 pairs of traits. Table 1 shows that the approximate version is much faster than either the complete version or any other software package tested. Detailed results are in Supplementary Figures S1–S4. In short, biMM and GEMMA gave essentially the same results and they were also similar to the results from GCTA and BOLT-REML.

**Table 1.** Cumulative run time in hours over 120 trait pairs of Figure 1

	biMM approx	biMM compl	GEMMA	BOLT-REML	GCTA
Real (h)	0.05	0.49	2.76	19.20	21.39
CPU (h)	0.07	1.49	2.76	19.20	21.39

‘Real’ is wall clock time. ‘CPU’ is total CPU time over all cores used.

To assess scaling to larger datasets, we evaluated how much time each additional trait pair would require for a dataset of 20 000 individuals after the eigendecomposition is available and phenotype data are completely observed. The resulting times in CPU seconds are 1.6 for biMM, 75 for GEMMA and 2670 for BOLT-REML. GCTA was unable to run with 16 Gb of RAM. The difference between biMM and GEMMA in this example with no missing data is that biMM holds the eigendecomposition in RAM while GEMMA reads it from a file for each pair of traits. The eigendecomposition itself took 70 CPU minutes with biMM and 450 CPU minutes with GEMMA. Hence, with a desktop computer, an analysis of completely observed or imputed omics data for 500 traits (124 750 trait pairs) measured on 20 000 individuals would take less than 2.5 days with biMM, over 100 days with GEMMA (although with a multivariate analysis strategy GEMMA could finish in 14 days, Supplementary Information) and many years with BOLT-REML.

## 4 Conclusion

Our freely available biMM software package makes a bivariate linear mixed model analysis of high-dimensional phenotypes on cohorts of a few tens of thousands of individuals practical using a desktop computer. For even larger cohorts, where explicit matrix decompositions are impractical on current desktop computers, BOLT-REML may be the only available option to analyze a pair of traits, but it cannot utilize sharing of individuals across trait pairs to efficiently analyze tens of thousands of trait pairs.

## Supplementary Material

Supplementary Data
