# Supplementary material for: biMM: efficient estimation of genetic variances and covariances for cohorts with high-dimensional phenotype measurements
Source: Bioinformatics. 2017 Mar 28;33(15):2405–7. doi: 10.1093/bioinformatics/btx166 (PMC5860115; doi:10.1093/bioinformatics/btx166)
Supplement: Supplementary Data [file btx166_supplementary_information.pdf]

biMM: Efficient estimation of genetic variances and covariances for cohorts with high-dimensional phenotype measurements

Supplementary information

Matti Pirinen et al.

February 8, 2017

Contents

S1 Data used in Example analysis **2**
S1.1 Northern Finland Birth Cohort 1966 (NFBC1966) 2
S1.2 Simulated data with 20,000 individuals 2

S2 Comparison of the methods **3**
S2.1 Northern Finland Birth Cohort 1966 (NFBC1966) 3
S2.2 Simulated data with 20,000 individuals 4

S3 Polygenic model for genome-wide data **6**
S3.1 Univariate polygenic model 7
S3.2 Bivariate polygenic model 8
S3.3 biMM algorithm 8
S3.3.1 Likelihood computation 9
S3.3.2 Ordering pairs, imputing and dropping values 12
S3.4 Testing biMM 12

S1 Data used in Example analysis

S1.1 Northern Finland Birth Cohort 1966 (NFBC1966)

NFBC1966 is a birth cohort study of children born in 1966 in the two northernmost provinces of Finland originally designed to focus on factors affecting pre-term birth, low birth weight, and subsequent morbidity and mortality [10]. The blood sample for the DNA extraction and all phenotype data used in the present study were collected at a follow-up visit when the participants were 31 years of age. The phenotypes (Table S1) were adjusted for sex and first ten principal components of genetic structure. Additionally, blood pressure measurements were adjusted for BMI. The phenotype data are the same as used by Tukiainen *et al.* [12].

Genotyping was done using Illumina 370K chip. We computed the genetic relationship matrix $\boldsymbol{R}$ by using $K = 319,445$ genotyped SNPs with minor-allele frequency (MAF) above 0.01.

ID	Trait	N	h^2_{ex}	SE_{ex}	h^2_{ap}	SE_{ap}
BMI	Body mass index	4843	0.277	0.056	0.277	0.056
CRP	C-reactive protein	4945	0.269	0.046	0.281	0.048
DBP	Diastolic blood pressure	4736	0.118	0.057	0.115	0.056
FGL	Fasting glucose	4820	0.165	0.053	0.164	0.053
HDL	HDL cholesterol	4843	0.300	0.055	0.307	0.056
HGT	Standing height	5025	0.614	0.053	0.610	0.055
HIP	Hip circumference	4814	0.185	0.058	0.187	0.058
FIN	Fasting insulin	4792	0.146	0.056	0.143	0.056
LDL	LDL cholesterol	4828	0.354	0.057	0.350	0.057
SBP	Systolic blood pressure	4743	0.127	0.057	0.137	0.056
TC	Total cholesterol	4843	0.279	0.056	0.277	0.056
TG	Triglyserides	4842	0.192	0.056	0.188	0.056
WHR	Waist-hip ratio	4814	0.102	0.053	0.098	0.053
WAI	Waist circumference	4815	0.140	0.055	0.140	0.055
WGT	Weight	4843	0.238	0.057	0.238	0.057
WAB	WHR adjusted for BMI	4812	0.082	0.054	0.080	0.053

Table S1: Sixteen quantitative traits analysed in NFBC1966 data. Heritability estimate (h^2) and its standard error (SE) are from biMM. Exact (ex) analysis was ran with $t_i = t_d = 0$ and approximate (ap) analysis was ran with $t_i = t_d = 200$. N is the number of individuals with a measurement for the trait.

S1.2 Simulated data with 20,000 individuals

We generated data for 20,000 individuals with HAPGEN2 using 117 Utah residents with Northern and Western European ancestry from the CEPH collection

(CEU panel) from HapMap3 (r2.b36) data on chromosome 2 (116,430 SNPs). We computed the genetic relationship matrix $\boldsymbol{R}$ by using $K = 98,516$ SNPs with minor-allele frequency (MAF) above 0.01. We generated 100 phenotype vectors with heritabilities between 0.2 and 0.8. The phenotype vectors were uncorrelated and contained no missing data.

S2 Comparison of the methods

We compared biMM 1.0.0, GEMMA 0.94.1 [19], BOLT-REML 2.2 with noRefine option for increased speed [8] and GCTA 1.25.3 [16]. We used a desktop computer with an Intel Quad-Core i7-3770 CPU 3.40 GHz and 16 Gb of RAM.

S2.1 Northern Finland Birth Cohort 1966 (NFBC1966)

Below we show the scatter plots for six pairs of the four methods: biMM (complete, $t_i = t_d = 0$), GEMMA, BOLT-REML and GCTA across 120 trait pairs reported in Example analysis of the main paper, for heritabilities V_G, genetic correlations ρ_G and their standard errors SE(V_G) and SE(ρ_G).

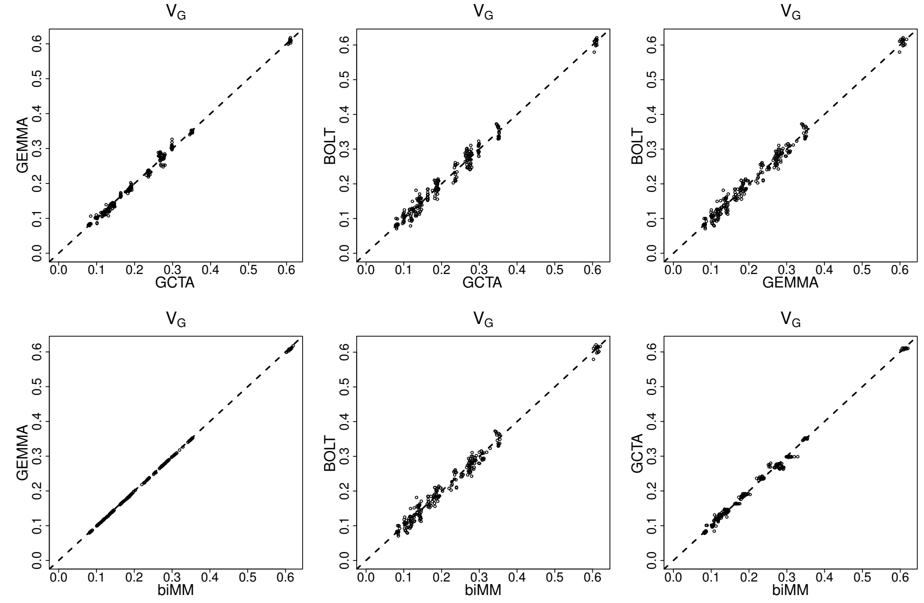

Figure S1: Heritability estimates across 120 pairs of traits from NFBC1966 data.

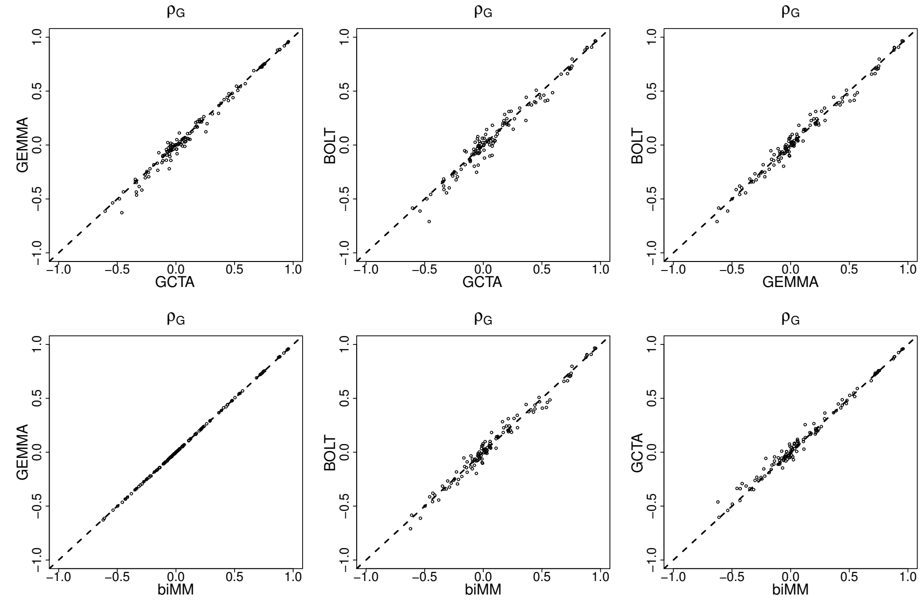

Figure S2: Genetic correlation estimates across 120 pairs of traits from NFBC1966 data.

S2.2 Simulated data with 20,000 individuals

We ran biMM, GEMMA and BOLT-REML on the data set with 20,000 individuals but were unable to run GCTA with 16 Gb of memory. We focused on the completely observed (or completely pre-imputed) data because there GEMMA's option to reuse an eigendecomposition can speed up the analysis. After an eigendecomposition for $\boldsymbol{R}$ matrix was done (70 CPU minutes with biMM and 450 CPU minutes with GEMMA), the run times per each additional pair of traits was 1.6 CPU seconds for biMM and 75 CPU seconds for GEMMA. BOLT-REML took 45 CPU minutes to analyse one pair of traits and does not benefit from a stored eigendecomposition. The difference between biMM and GEMMA is mainly that GEMMA reads in the eigendecomposition from a file for each pair of analyzed traits whereas biMM is able to hold it in RAM. Next, we evaluated whether the analysis using GEMMA or BOLT-REML could be sped up by analyzing more than two traits at a time.

Let us assume that we have T traits, i.e. $T(T-1)/2$ trait pairs, to analyze and that we use GEMMA or BOLT-REML by applying it to t traits at a time. This can be done by first analyzing $B = T/t$ non-ovelapping blocks of the traits (block 1: $1,\ldots,t$; block 2: $t+1,\ldots,2t$ etc.) and then by computing the covariances for the remaining pairs by applying GEMMA/BOLT-REML four times per each pair of blocks. For example, to compute the cross-covariances between blocks 1 and 2, we do the following four analyses, each having exactly t traits: $(1,\ldots,t/2,t+1,\ldots,3t/2)$, $(1,\ldots,t/2,3t/2+1,\ldots,2t)$, $(t/2+1,\ldots,t,t+$

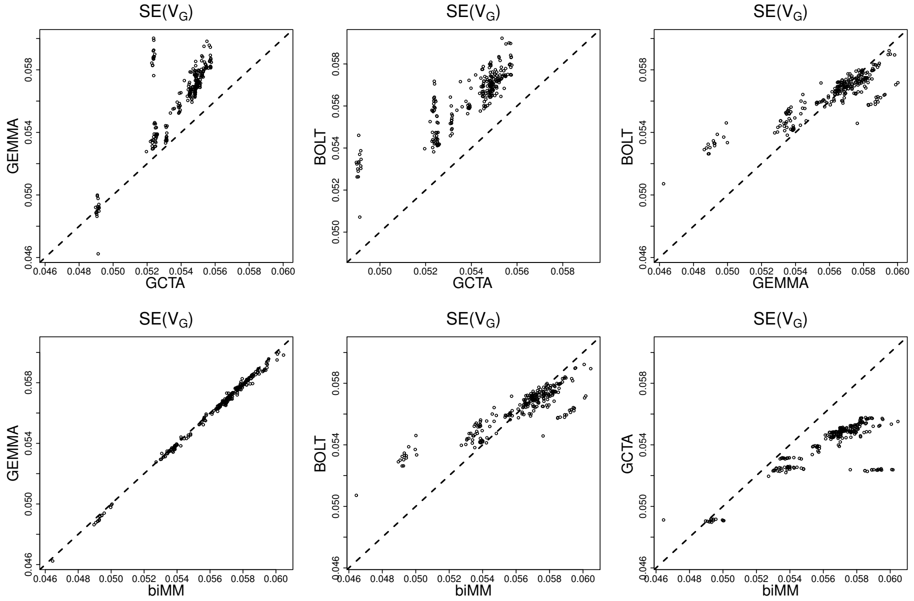

Figure S3: Estimated standard errors of heritability estimates across 120 pairs of traits from NFBC1966 data.

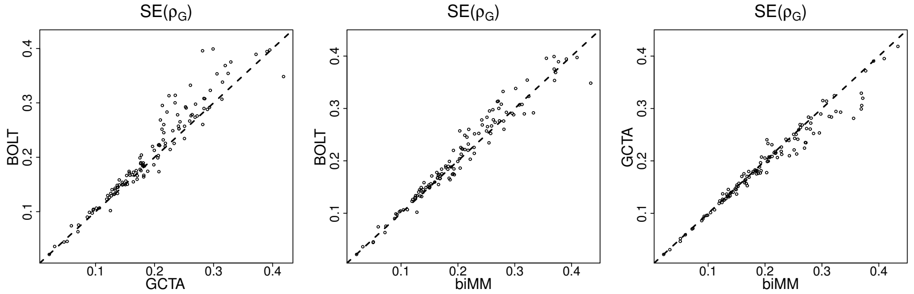

Figure S4: Estimates of standard errors of genetic correlation estimates across 120 pairs of traits from NFBC1966 data. Not available for GEMMA.

$1, \ldots, 3t/2)$ and $(t/2+1, \ldots, t, 3t/2+1, \ldots, 2t)$. Thus, altogether we need $B + 4\binom{B}{2} = B(2B-1)$ applications of t dimensional analysis.

Table S2 reports run time of GEMMA and BOLT-REML for $t = 2, 4, \ldots, 20$ dimensional analyses as well as an expectetd run time to analyze $T = 500$ traits, i.e., 124,750 trait pairs. We see that a multi-trait analysis strategy can drop the total run time from 100 days to 14 days with GEMMA and from 11 years to 7 years with BOLT-REML. For both methods, there seems to be an optimal number of traits per analysis (6-8 with GEMMA, 4-6 with BOLT-REML) after which an increase in the number of traits also increases the total run time.

GEMMA								
t	2	4	6	8	10	12	16	20
one (min)	1.25	1.28	1.57	2.68	5.77	13.70	74.06	322.68
all (day)	104	27	15	14	20	33	99	275
BOLT-REML								
t	2	4	6	8	10	12	16	20
one (min)	45	115	270	667	-	-	-	-
all (year)	10.7	6.8	7.1	9.8	-	-	-	-

Table S2: Run times of GEMMA and BOLT-REML for $t = 2, 4, \ldots, 20$ dimensional analysis for one data set with 20,000 individuals in CPU minutes (row 'one') and for all pairs of $T = 500$ traits in CPU days or CPU years (row 'all'). For GEMMA, eigendecomposition is read from a file. Run time was not measured for BOLT-REML with $t > 8$.

S3 Polygenic model for genome-wide data

To derive the linear mixed model, we consider a polygenic model that assumes that the genetic component of the traits is distributed among a large number of individual variants having additive effects. When applied to "unrelated" individuals (or more precisely, only very distantly related individuals from our population cohorts), the model decomposes the trait variance into an additive genetic component (G) that is due to the available panel of SNPs, and the environmental component (ε) that includes also higher order genetic components together with the part of the additive component that is not captured by G. In particular, the bivariate model can be used for estimating a lower bound for the additive genetic variance for both phenotypes, the correlation between the additive genetic components of the two phenotypes and the correlation between the environmental components of the two phenotypes.

For large-scale human genetic studies with a univariate phenotype, the corresponding model was introduced by Yang et al. in their software package GCTA [15] and further explained by Visscher et al. [13] and Zaitlen and Kraft [17]. The bivariate extension was recently considered by Deary et al. [4], Korte et al. [6] and Davis et al. [3], and an extension to more than two traits by the GEMMA algorithm of Zhou and Stephens [19] and BOLT-REML by Loh et al. [8]. A completely new way to estimate some of the parameters of the univariate and bivariate polygenic model via LD-score regression using summary statistics from genome-wide association studies was recently intoduced by Bulik-Sullivan et al. [2, 1].

Next we formulate the univariate (section 3.1) and bivariate (section 3.2) versions of the model, explain how we do the computation efficiently (section 3.3) and give simulation results to verify that we have a valid interpretation of the parameter estimates (section 3.4).

S3.1 Univariate polygenic model

We start by describing the model for a univariate phenotype (say phenotype 1). Let $\boldsymbol{Y}_1$ be a vector of observed quantitative measurements for n individuals. Let g^*_{ik} be the standardized genotype of individual i at SNP k, that is,

$$g^*_{ik} = \frac{g_{ik} - 2\widehat{f}_k}{\sqrt{2\widehat{f}_k\left(1-\widehat{f}_k\right)}}\ ,$$

where g_{ik} is the minor allele count (0,1 or 2) that i carries at locus k and $\widehat{f}_k = \frac{1}{2n}\sum_{i=1}^{n} g_{ik}$ is the sample allele frequency. The linear model assumes that

$$y_{i1} = \mu_{i1} + \sum_k g^*_{ik}\beta_{k1} + \varepsilon_{i1}, \tag{S1}$$

where β_{k1} is the effect of SNP k on Y_1, ε_{i1} is the environmental term of Y_1 for individual i and the sum is over all the SNPs. The term μ_{i1} describes the expected phenotype of individual i after all the non-genetic covariates (such as age, sex and cohort) have been taken into account, for example, by considering residuals from a regression model.

Following [15], we assume that $\beta_{k1} \sim \mathcal{N}(0, v_{g1})$ for each SNP k and $\varepsilon_{i1} \sim \mathcal{N}(0, V_{\varepsilon 1})$ for each individual i. Then the additive genetic variance of Y_1 due to the SNPs,

$$V_{G1} = \mathrm{Var}\left(\sum_{k=1}^{K} g^*_{ik}\beta_{k1}\right) \approx \sum_{k=1}^{K}\mathrm{Var}\left(g^*_{ik}\beta_{k1}\right) = K v_{g1},$$

can be estimated by a linear mixed model formulation of the model (S1) [15, 17]:

$$\boldsymbol{Y}_1 = \boldsymbol{\mu}_1 + \boldsymbol{G}_1 + \boldsymbol{\varepsilon}_1, \tag{S2}$$

where $\boldsymbol{\varepsilon}_1 \sim \mathcal{N}(0, V_{\varepsilon 1}\boldsymbol{I})$ and $\boldsymbol{G}_1 \sim \mathcal{N}(0, V_{G1}\boldsymbol{R})$ with the element $\boldsymbol{R}_{ij}$ of the matrix $\boldsymbol{R}$ being

$$\boldsymbol{R}_{ij} = \frac{1}{K}\sum_k g^*_{ik}g^*_{jk} = \frac{1}{K}\sum_k \frac{\left(g_{ik} - 2\widehat{f}_k\right)\left(g_{jk} - 2\widehat{f}_k\right)}{2\widehat{f}_k\left(1-\widehat{f}_k\right)}.$$

Note that since the variance of the effect size distribution, v_{g1}, is the same for all standardized SNPs, it follows that the variance of the effect size distribution for an allele at SNP k, $v_{g1}/\left(2\widehat{f}_k\left(1-\widehat{f}_k\right)\right)$, grows as the minor allele frequency $\widehat{f}_k$ decreases. Thus, this model assumes larger per allele effect sizes at rarer SNPs than at more common SNPs.

S3.2 Bivariate polygenic model

We follow the extension of the linear model to the bivariate case given by [4]. This corresponds to the model $\boldsymbol{Y} = \boldsymbol{\mu} + \boldsymbol{G} + \boldsymbol{\varepsilon}$, where

$$\boldsymbol{Y} = \begin{bmatrix} \boldsymbol{Y}_1 \\ \boldsymbol{Y}_2 \end{bmatrix}, \quad \boldsymbol{\mu} = \begin{bmatrix} \boldsymbol{\mu}_1 \\ \boldsymbol{\mu}_2 \end{bmatrix}, \quad \boldsymbol{G} \sim \mathcal{N}(0, \boldsymbol{\Sigma}_G), \text{ and } \boldsymbol{\varepsilon} \sim \mathcal{N}(0, \boldsymbol{\Sigma}_\varepsilon), \tag{S3}$$

with

$$\begin{aligned} \boldsymbol{\Sigma}_G &= \left[\begin{array}{c|c} V_{G1}\boldsymbol{R} & V_{G12}\boldsymbol{R} \\ \hline V_{G12}\boldsymbol{R} & V_{G2}\boldsymbol{R} \end{array}\right] \text{ and} \\ \boldsymbol{\Sigma}_\varepsilon &= \left[\begin{array}{c|c} V_{\varepsilon 1}\boldsymbol{I} & V_{\varepsilon 12}\boldsymbol{I} \\ \hline V_{\varepsilon 12}\boldsymbol{I} & V_{\varepsilon 2}\boldsymbol{I} \end{array}\right] \end{aligned}$$

expressed as $n \times n$ blocks, where n is the number of individuals.

From this model, an estimate of V_{Gt} gives a lower bound for the additive genetic variance of each trait ($t = 1, 2$) and thus can be used to estimate a lower bound for the (narrow-sense) heritability. We can also estimate the genetic correlation $\rho_G = V_{G12}/\sqrt{V_{G1}V_{G2}}$ which can be interpreted as the correlation of the additive genetic components. Similarly we can estimate $\rho_\varepsilon = V_{\varepsilon 12}/\sqrt{V_{\varepsilon 1}V_{\varepsilon 2}}$, the correlation in the environmental terms between the phenotypes.

At the level of individual genetic effects, this model corresponds to the assumption that for all $k \leq K$

$$\begin{bmatrix} \boldsymbol{\beta}_{k1} \\ \boldsymbol{\beta}_{k2} \end{bmatrix} \sim \mathcal{N}_2 \left(0, \begin{bmatrix} v_{g1} & v_{g12} \\ v_{g12} & v_{g2} \end{bmatrix} \right), \tag{S4}$$

where v_{gt} is the variance of the individual genetic effect on trait $t = 1, 2$ and v_{g12} is the covariance between the effects of a single variant on the two traits. We will consider the consequences of this assumption below at section S3.4.

S3.3 biMM algorithm

Our goal is to make an efficient algorithm for estimating (co)variance parameters in the setting where hundreds of traits have been measured on the same set of individuals. For this purpose, we extend computational solutions of previous algorithms on a univariate linear mixed model [18, 7, 9] to the bivariate case. The biMM algorithm described here has not been published before although M Pirinen has applied some ideas of biMM's likelihood computation in an earlier study of reading and mathematics skills [3].

Recently, general algorithms for multivariate linear mixed models have been introduced by Zhou and Stephens [19] and Loh et al. [8] with applications to

genome-wide data sets.We see two main contributions of the biMM algorithm given the existing work in the field: (1) a new and simple way to write down the likelihood function of bivariate LMM using $\mathcal{O}(n)$ operations; and (2) implementation that is particularly efficient for estimating pairwise variance components across thousands of trait pairs including the functionality to order traits suitably and to impute or to ignore only some trait values as necessary for efficient computation.

While an appropriately adjusted version of the GEMMA algorithm of Zhou and Stephens [19] might also lead to a quick bivariate likelihood computation, to our knowledge, no publicly available implementation is available to efficiently handle our setting of hundreds of traits with possibly missing values.

BOLT-REML is the only existing linear mixed model implementation that can run on very large numbers of individuals (Loh et al. reported test runs with 50,000 [8]). Since BOLT-REML bypasses the generation of the explicit relationship matrix, it is based on very different approximations compared to biMM, GEMMA or GCTA. The differences in heritability estimates between the methods were a bit larger between BOLT-REML and the other methods (Figure S1) than among the other methods themselves. Additionally, BOLT-REML is much slower than biMM or GEMMA with cohort sizes between 5,000 and 20,000 individuals tested in the main paper. Thus, while BOLT-REML importantly extends the range of linear mixed model computations to very large cohorts, there are good reasons to still consider the approach based on explicit matrix computations when those are feasible to carry out, i.e., when the cohort size is up to a few tens of thousands of individuals.

S3.3.1 Likelihood computation

The log-likelihood function for model (S3), after the covariates and the population mean in $\boldsymbol{\mu}$ have been regressed out from $\boldsymbol{Y}$, is

$$L(\boldsymbol{V}) = -n\log(2\pi) - \frac{1}{2}\log(|\boldsymbol{\Sigma}|) - \frac{1}{2}\boldsymbol{Y}^T\boldsymbol{\Sigma}^{-1}\boldsymbol{Y}, \tag{S5}$$

where variance parameters $\boldsymbol{V} = (V_{G1}, V_{G2}, V_{G12}, V_{\varepsilon 1}, V_{\varepsilon 2}, V_{\varepsilon 12})$ are included in the matrix $\boldsymbol{\Sigma} = \boldsymbol{\Sigma}_G + \boldsymbol{\Sigma}_\varepsilon$, and $|\boldsymbol{\Sigma}|$ denotes the determinant of $\boldsymbol{\Sigma}$.

The eigenvalue decomposition of the positive semi-definite matrix $\boldsymbol{R}$ yields an orthonormal $n \times n$-matrix $\boldsymbol{U}$ of eigenvectors and a diagonal $n \times n$-matrix $\boldsymbol{D}$ of non-negative eigenvalues for which $\boldsymbol{R} = \boldsymbol{U}\boldsymbol{D}\boldsymbol{U}^T$ (see e.g. [5]). Because $\boldsymbol{U}\boldsymbol{U}^T = \boldsymbol{I}$ (orthonormality) it follows that

$$\begin{aligned}
\boldsymbol{\Sigma} &= \left[\begin{array}{c|c} V_{G1}\boldsymbol{R} + V_{\varepsilon 1}\boldsymbol{I} & V_{G12}\boldsymbol{R} + V_{\varepsilon 12}\boldsymbol{I} \\ \hline V_{G12}\boldsymbol{R} + V_{\varepsilon 12}\boldsymbol{I} & V_{G2}\boldsymbol{R} + V_{\varepsilon 2}\boldsymbol{I} \end{array}\right] \\
&= \left[\begin{array}{c|c} \boldsymbol{U} & \boldsymbol{0} \\ \hline \boldsymbol{0} & \boldsymbol{U} \end{array}\right] \left[\begin{array}{c|c} V_{G1}\boldsymbol{D} + V_{\varepsilon 1}\boldsymbol{I} & V_{G12}\boldsymbol{D} + V_{\varepsilon 12}\boldsymbol{I} \\ \hline V_{G12}\boldsymbol{D} + V_{\varepsilon 12}\boldsymbol{I} & V_{G2}\boldsymbol{D} + V_{\varepsilon 2}\boldsymbol{I} \end{array}\right] \left[\begin{array}{c|c} \boldsymbol{U}^T & \boldsymbol{0} \\ \hline \boldsymbol{0} & \boldsymbol{U}^T \end{array}\right] \\
&= \left[\begin{array}{c|c} \boldsymbol{U} & \boldsymbol{0} \\ \hline \boldsymbol{0} & \boldsymbol{U} \end{array}\right] \left[\begin{array}{c|c} \boldsymbol{\Delta}(a_{i1}) & \boldsymbol{\Delta}(a_{i12}) \\ \hline \boldsymbol{\Delta}(a_{i12}) & \boldsymbol{\Delta}(a_{i2}) \end{array}\right] \left[\begin{array}{c|c} \boldsymbol{U}^T & \boldsymbol{0} \\ \hline \boldsymbol{0} & \boldsymbol{U}^T \end{array}\right],
\end{aligned}$$

where $\mathbf{\Delta}(a_{i1})$ is the diagonal matrix whose diagonal elements are $a_{11}, \ldots, a_{n1}$ and we have used notation

$$\begin{aligned} a_{i1} &= V_{G1} d_i + V_{\varepsilon 1} \\ a_{i2} &= V_{G2} d_i + V_{\varepsilon 2} \\ a_{i12} &= V_{G12} d_i + V_{\varepsilon 12}, \end{aligned}$$

where d_i is the ith eigenvalue of $\boldsymbol{R}$, that is, the element (i,i) of $\boldsymbol{D} = \mathbf{\Delta}(d_i)$.

To compute the determinant, we use the fact [11] that if $\boldsymbol{B}_3\boldsymbol{B}_4 = \boldsymbol{B}_4\boldsymbol{B}_3$ for a block matrix

$$\boldsymbol{B} = \left[\begin{array}{c|c} \boldsymbol{B}_1 & \boldsymbol{B}_2 \\ \hline \boldsymbol{B}_3 & \boldsymbol{B}_4 \end{array}\right], \text{ then } \det(\boldsymbol{B}) = \det(\boldsymbol{B}_1\boldsymbol{B}_4 - \boldsymbol{B}_2\boldsymbol{B}_3).$$

$$\begin{aligned} \det(\mathbf{\Sigma}) &= \det(\boldsymbol{U}\boldsymbol{U} - \mathbf{0}\mathbf{0}) \\ &\times \det\left(\mathbf{\Delta}(a_{i1})\mathbf{\Delta}(a_{i2}) - \mathbf{\Delta}(a_{i12})\mathbf{\Delta}(a_{i12})\right) \\ &\times \det(\boldsymbol{U}^T\boldsymbol{U}^T - \mathbf{0}\mathbf{0}) \\ &= 1 \times \prod_{i=1}^{n} \left(a_{i1}a_{i2} - a_{i12}^2\right) \times 1 \\ &= \prod_{i=1}^{n} c_i, \end{aligned}$$

where

$$c_i = a_{i1}a_{i2} - a_{i12}^2, \text{ for } i = 1, \ldots, n.$$

To compute the inverse $\mathbf{\Sigma}^{-1}$ we use a formula for block matrices:

$$\left[\begin{array}{c|c} \boldsymbol{B}_1 & \boldsymbol{B}_2 \\ \hline \boldsymbol{B}_3 & \boldsymbol{B}_4 \end{array}\right]^{-1} = \left[\begin{array}{c|c} \left(\boldsymbol{B}_1 - \boldsymbol{B}_2\boldsymbol{B}_4^{-1}\boldsymbol{B}_3\right)^{-1} & -\left(\boldsymbol{B}_1 - \boldsymbol{B}_2\boldsymbol{B}_4^{-1}\boldsymbol{B}_3\right)^{-1}\boldsymbol{B}_2\boldsymbol{B}_4^{-1} \\ \hline -\left(\boldsymbol{B}_4 - \boldsymbol{B}_3\boldsymbol{B}_1^{-1}\boldsymbol{B}_2\right)^{-1}\boldsymbol{B}_3\boldsymbol{B}_1^{-1} & \left(\boldsymbol{B}_4 - \boldsymbol{B}_3\boldsymbol{B}_1^{-1}\boldsymbol{B}_2\right)^{-1} \end{array}\right].$$

By applying this to matrix

$$\boldsymbol{A} = \left[\begin{array}{c|c} \mathbf{\Delta}(a_{i1}) & \mathbf{\Delta}(a_{i12}) \\ \hline \mathbf{\Delta}(a_{i12}) & \mathbf{\Delta}(a_{i2}) \end{array}\right],$$

we have that

$$\boldsymbol{A}^{-1} = \left[\begin{array}{c|c} \mathbf{\Delta}(\frac{1}{c_i}) & \mathbf{0} \\ \hline \mathbf{0} & \mathbf{\Delta}(\frac{1}{c_i}) \end{array}\right] \left[\begin{array}{c|c} \mathbf{\Delta}(a_{i2}) & \mathbf{\Delta}(-a_{i12}) \\ \hline \mathbf{\Delta}(-a_{i12}) & \mathbf{\Delta}(a_{i1}) \end{array}\right].$$

By transformation

$$\widetilde{\boldsymbol{Y}} = \left[\begin{array}{c|c} \boldsymbol{U}^T & \mathbf{0} \\ \hline \mathbf{0} & \boldsymbol{U}^T \end{array}\right] \boldsymbol{Y}$$

the log-likelihood (S5) becomes

$$
\begin{aligned}
L(\boldsymbol{V}) &= -n\log(2\pi) - \frac{1}{2}\log(|\boldsymbol{\Sigma}|) - \frac{1}{2}\boldsymbol{Y}^T\boldsymbol{\Sigma}^{-1}\boldsymbol{Y} \\
&= -n\log(2\pi) - \frac{1}{2}\sum_{i=1}^{n}\log(c_i) - \frac{1}{2}\widetilde{\boldsymbol{Y}}^T\boldsymbol{A}^{-1}\widetilde{\boldsymbol{Y}} \\
&= -n\log(2\pi) - \frac{1}{2}\sum_{i=1}^{n}\log(c_i) - \frac{1}{2}\sum_{i=1}^{n}\frac{\widetilde{y_{i1}}^2 a_{i2} + \widetilde{y_{i2}}^2 a_{i1} - 2\widetilde{y_{i1}}\widetilde{y_{i2}}a_{i12}}{c_i}.
\end{aligned}
$$

For each set of values of the parameters $\boldsymbol{V}$, the evaluation of the log-likelihood requires $\mathcal{O}(n)$ basic operations, where n is the number of individuals. Note that a naive evaluation of the likelihood would require $\mathcal{O}(n^3)$ operations for each set of parameters due to computational complexity of the determinant and matrix inversion.

To optimize the likelihood we use a combination of Nelder-Mead and BFGS algorithms as implemented in the optim function of the R software package. For BFGS we need the gradient of the log-likelihood.

Derivatives

With the notation defined above we have:

$$
\begin{aligned}
\frac{\partial L}{\partial V_{G1}} &= -\frac{1}{2}\sum_{i=1}^{n}\frac{d_i c_i\left(a_{i2} + \widetilde{y_{i2}}^2\right) - d_i a_{i2} b_i}{c_i^2} \\
\frac{\partial L}{\partial V_{G2}} &= -\frac{1}{2}\sum_{i=1}^{n}\frac{d_i c_i\left(a_{i1} + \widetilde{y_{i1}}^2\right) - d_i a_{i1} b_i}{c_i^2} \\
\frac{\partial L}{\partial V_{\varepsilon 1}} &= -\frac{1}{2}\sum_{i=1}^{n}\frac{c_i\left(a_{i2} + \widetilde{y_{i2}}^2\right) - a_{i2} b_i}{c_i^2} \\
\frac{\partial L}{\partial V_{\varepsilon 2}} &= -\frac{1}{2}\sum_{i=1}^{n}\frac{c_i\left(a_{i1} + \widetilde{y_{i1}}^2\right) - a_{i1} b_i}{c_i^2} \\
\frac{\partial L}{\partial V_{G12}} &= -\frac{1}{2}\sum_{i=1}^{n}\frac{-2d_i c_i\left(a_{i12} + \widetilde{y_{i1}}\widetilde{y_{i2}}\right) + 2d_i a_{i12} b_i}{c_i^2} \\
\frac{\partial L}{\partial V_{\varepsilon 12}} &= -\frac{1}{2}\sum_{i=1}^{n}\frac{-2c_i\left(a_{i12} + \widetilde{y_{i1}}\widetilde{y_{i2}}\right) + 2a_{i12} b_i}{c_i^2}
\end{aligned}
$$

where

$$
b_i = \widetilde{y_{i1}}^2 a_{i2} + \widetilde{y_{i2}}^2 a_{i1} - 2\widetilde{y_{i1}}\widetilde{y_{i2}}a_{i12}, \text{ for } i = 1, \ldots, n.
$$

S3.3.2 Ordering pairs, imputing and dropping values

To make eigendecomposition as reusable as possible biMM orders the trait pairs (or traits in the univariate case) to maximize the sample overlap between consecutive pairs.

Given the first pair, we greedily choose the next pair from among the pairs having the smallest distance to the current pair. Here the distance between the pairs means the number of individuals for which one of the trait pairs has complete data and the other pair does not have complete data. After the second pair is chosen, the same process continues through the subsequent trait pairs until the full ordering is determined. If the first pair is not given, biMM iterates over all possible starting values and chooses the one that leads to the smallest number of eigendecompositions given the parameters t_i and t_d.

The input values t_i and t_d determine, respectively, for how many individuals biMM is allowed to impute the missing trait values and for how many individuals biMM is allowed to set the trait values missing, i.e., to drop the individuals from the analysis. A new eigendecomposition will be made if and only if the analysis of the next trait / trait pair would require imputing more than t_i individuals or dropping more than t_d individuals.

For the trait imputation, the user specifies k_{imp}, i.e., the number of the traits that are used for imputing the missing values. When imputing trait T, for each individual with a missing value, biMM chooses those k_{imp} traits that are most correlated (in absolute value) with trait T in the whole cohort and are observed for the individual considered. Trait T is imputed as the expected value from a conditional distribution of trait T given the observed value of the k_{imp} other traits and an assumption that their $k_{imp} + 1$ dimensional joint distribution is a multivariate Gaussian. The default value $k_{imp} = 0$ corresponds to the mean value imputation.

S3.4 Testing biMM

Assume that there are S shared variants that affect both traits and T_1 and T_2 variants that affect only trait 1 and trait 2, respectively. Assume further that the effect sizes for all these variants follow the normal distribution with zero mean and variances v_{g1} for trait 1 and v_{g2} for trait 2, and that for each shared variant j, $\mathrm{cor}(\beta_{j1}, \beta_{j2}) = \rho_S$. Then the covariance between the genetic components of individual i is

$$\mathrm{Cov}(G_{i1}, G_{i2}) = S\rho_S\sqrt{v_{g1}v_{g2}}$$

and the genetic variances are $V_{G1} = (S + T_1)v_{g1}$ and $V_{G2} = (S + T_2)v_{g2}$. It follows that the genetic correlation is

$$\rho_G = \frac{S\rho_S}{\sqrt{S+T_1}\sqrt{S+T_2}} = \frac{\rho_S}{\sqrt{1+T_1/S}\sqrt{1+T_2/S}}. \tag{S6}$$

In particular, if $T_1 = T_2 = 0$ (all variants shared), then $\rho_G = \rho_S$, but if $\max\{T1, T2\} \gg S$ (most variants trait-specific), then $\rho_G \approx 0$, independent

Scenario	V_{G1}	V_{G2}	S	T_1	T_2	ρ	ρ_S	ρ_G	ρ_ε
1	0.2	0.5	200	200	800	0.5	0.8	0.25	0.66
2	0.2	0.5	200	200	800	-0.4	0.8	0.25	-0.76
3	0.2	0.5	400	0	600	0.4	-0.6	-0.38	0.82
4	0.3	0.8	100	100	1900	0.0	0.6	0.09	-0.12
5	0.2	0.3	2	198	1998	-0.5	0.0	0	-0.67
6	0.2	0.5	1000	1000	1000	0.0	0.8	0.4	-0.2

Table S3: Parameters for data simulation. V_{Gt} is the total genetic variance for trait $t = 1, 2$, S is the number of shared variants, T_t is the number of variants specific to trait $t = 1, 2$, ρ is the total correlation between the traits, ρ_S is the correlation of the effect sizes of the shared variants, ρ_G is the genetic correlation between the traits, ρ_ε is the environmental correlation between the traits.

of ρ_S. In general, the genetic correlation ρ_G is an average property of all variants affecting the traits that results from shrinking the shared correlation ρ_S by (harmonic mean of) the proportion of the shared genetic effects.

We hope that by the linear mixed model we could get unbiased estimates of genetic variances V_{G1} and V_{G2} as well as correlation parameters ρ_G and ρ_ε. However, this is not self-evident, because the model makes an assumption that every one of the K variants in the genome comes from the non-zero bivariate effect size distribution, which is clearly violated whenever only a minority of the variants truly have a non-zero effect. Thus, to validate our interpretation of the variance parameters in a realistic scenarios, and to verify our implementation of biMM, we did some simulation studies. Recently, some theoretical conditions for unbiasedness of univariate variance parameters have been listed by Yang et al. [14].

Simulations: We chose 5,000 individuals from the NFBC1966 data and used the $\boldsymbol{R}$ matrix of $K = 319,445$ genotyped SNPs with minor-allele frequency (MAF) above 0.01. We further extracted from the total set of SNPs a subset of 20,000 approximately equally spaced SNPs from which we picked those that truly affected the phenotypes. We generated 1,000 data sets under each of the six scenarios by varying values for the parameters at columns 2 to 8 in Table S3, which uniquely determine ρ_G and ρ_ε given in the last two columns of Table S3. We always kept the total variance of both traits at 1 and sampled the effect sizes for the variants from the Gaussian distribution with variance $V_{Gt}/(S+T_t)$ for trait t, and with the correlation ρ_S for the shared variants.

Results: The distribution of the maximum likelihood estimates over the 1,000 data sets for four parameters V_{G1}, V_{G2}, ρ_G and ρ_ε are given in Figure S5. Figure S5 shows that biMM gives (nearly) unbiased estimates of the parameters in all six scenarios that vary in the heritability, in the sign and magnitude of the correlations as well as in the proportion of the variants shared.

These results give confidence that we can interpret the estimates from biMM according to the heritability and correlation parameters of the polygenic model,

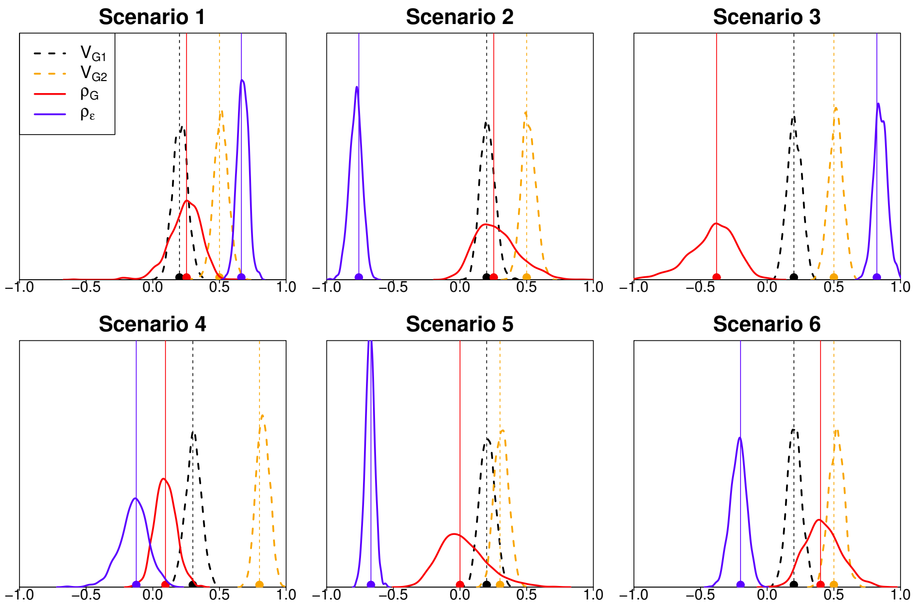

Figure S5: Distribution of maximum likelihood estimates for four parameters (legend top-left) from biMM across 1,000 simulated data sets for each of the six scenarios of Table S3. The vertical lines show the values used in the simulation.

even when the polygenic assumption is violated by a large majority of the variants having no effect on the traits at least as long as the variants with effects are a random sample from all variants included in the model.

The second derivatives allow analytic estimation of standard error for each parameter. If the sampling distribution of the parameter estimate were Gaussian, then the standard error would estimate the standard deviation of that sampling distribution. Table S4 shows empirical standard deviation of the parameter estimates over the simulations together with the median of the standard errors over the same simulations. In general, the results show that the estimated standard error gives a good estimate of the magnitude of the empirical standard deviation. It seems that the standard errors typically underestimate slightly the standard deviations for the correlation parameters and for smaller heritability values (V_{G1}) whereas for larger heritability values (V_{G2}) the standard errors overestimate slightly the standard deviations. We note that the sampling distributions for these parameters might not follow a Gaussian near the boundary values of the parameter (0 for variances, and ± 1 for correlations) and the interpretation of standard error is not as straightforward there.

	V_{G1}		V_{G2}		ρ_G		ρ_ε	
Scenario	SD	SE	SD	SE	SD	SE	SD	SE
1	0.055	0.053	0.055	0.056	0.12	0.11	0.042	0.041
2	0.055	0.052	0.053	0.055	0.16	0.15	0.048	0.046
3	0.053	0.052	0.052	0.055	0.17	0.16	0.05	0.046
4	0.059	0.054	0.050	0.057	0.08	0.079	0.11	0.11
5	0.056	0.053	0.054	0.054	0.18	0.16	0.035	0.034
6	0.054	0.053	0.056	0.056	0.13	0.13	0.06	0.061

Table S4: Uncertainty of parameters for data simulation. V_{Gt} is the total genetic variance for trait $t = 1, 2$, ρ_G is the genetic correlation between the traits, ρ_ε is the environmental correlation between the traits. For each scenario, SD is the empirical standard deviation of the point estimates and SE is the median of the analytically calculated standard errors.

References

[1] BK Bulik-Sullivan, HK Finucane, V Anttila, A Gusev, FD Day, PR Loh, ReproGen Consortium, Psychiatric Genomics Consortium, Genetic Consortium for Anorexia Nervosa of the Wellcome Trust Case Control Consortium 3, L Duncan, JRB Perry, N Patterson, EB Robinson, MJ Daly, AL Price, and BM Neale. An atlas of genetic correlations across human diseases and traits. *Nat Gen*, 47:1236–1241, 2015.

[2] BK Bulik-Sullivan, PR Loh, HK Finucane, S Ripke, J Yang, Schizophrenia Working Group of the Psychiatric Genomics Consortium, N Patterson, MJ Daly, AL Price, and BM Neale. LD Score regression distinguishes confounding from polygenicity in genome-wide association studies. *Nat Gen*, 47:291–295, 2015.

[3] OSP Davis, G Band, M Pirinen, CMA Haworth, EL Meabury, Y Kovas, N Harlaar, SJ Docherty, KB Hanscombe, M Trzaskowski, CJ C Curtis, A Strange, C Freeman, C Bellenguez, Z Su, R Pearson, D Vukcevic, C Langford, P Deloukas, S Hunt, E Gray, S Dronov, SC Potter, A Tashakkori-Ghanbaria, S Edkins, JM Blackwell SJ Bumpstead, E Bramon, MA Brown, JP Casas, A Corvin, A Duncanson, JAZ Jankowski, HS Markus, CG Mathew, CNA Palmer, A Rautanen, SJ Sawcer, RC Trembath, AC Viswanathan, NW Wood, I Barroso, L Peltonen, PS Dale, SA Petrill, LS Schalkwyk, IW Craig, CM Lewis, TS Price, P Donnelly The Wellcome Trust Case Control Consortium, R Plomin, and CCA Spencer. The correlation between reading and mathematics ability at age twelve has a substantial genetic component. *Nat Commun*, 5:4204, 2014.

[4] IJ Deary, J Yang, G Davies, SE Harris, A Tenesa, D Liewald, M Luciano, LM Lopez, AJ Gow, J Corley, P Redmond, HC Fox, SJ Rowe, P Haggarty, G McNeill, ME Goddard, DJ Porteous, LJ Whalley, JM Starr, and

PM Visscher. Genetic contributions to stability and change in intelligence from childhood to old age. *Nature*, 482:212–215, 2012.

[5] GH Golub and CF Van Loan. *Matrix Computations.* The Johns Hopkins University Press, Baltimore, USA, 3rd edition, 1996.

[6] A Korte, BJ Vilhjalmsson, V Segura, A Platt, Q Long, and M Nordborg. A mixed-model approach for genome-wide association studies of correlated traits in structured populations. *Nat Gen*, 44:1066–1071, 2012.

[7] C Lippert, J Listgarten, Y Liu, C M Kadie, R I Davidson, and D Heckerman. FaST linear mixed models for genome-wide association studies. *Nat Methods*, 8:833–835, 2012.

[8] PR Loh, G Bhatia, A Gusev, HK Finucane, BK Bulik-Sullivan, SJ Pollack, Schizophrenia Working Group of the Psychiatric Genomics Consortium, TR de Candia, SH Lee, NR Wray, KS Kendler, MC O'Donovan, BM Neale, N Patterson, and AL Price. Contrasting genetic architectures of schizophrenia and other complex diseases using fast variance-components analysis. *Nat Gen*, 47:1385–1392, 2015.

[9] M Pirinen, P Donnelly, and C Spencer. Efficient computation with a linear mixed model on large-scale data sets with applications to genetic studies. *Ann Appl Stat*, 7:369–390, 2013.

[10] P Rantakallio. Groups at risk in low birth weight infants and perinatal mortality. *Acta Paediatr Scand*, 193:191, 1969.

[11] JR Silvester. Detrminants of block matrices. *Math Gazette*, 10:460–467, 2000.

[12] T Tukiainen, M Pirinen, AP Sarin, C Ladenvall, Jo Kettunen, T Lehtimäki, ML Lokki, M Perola, J Sinisalo, E Vlachopoulou, JG Eriksson, L Groop, A Jula, MR Järvelin, OT Raitakari, V Salomaa, and S Ripatti. Chromosome x-wide association study identifies loci for fasting insulin and height and evidence for incomplete dosage compensation. *PLoS Gen*, 10:e1004127, 2014.

[13] PM Visscher, J Yang, and ME Goddard. A commentary on "Common SNPs explain a large proportion of the heritability for human height" by Yang et al. (2010). *Twin Research and Human Genetics*, 13:517–524, 2010.

[14] J Yang, A Bakshi, Z Zhu, G Hemani, AAE Vinkhuyzen, SH Lee, MR Robinson, JRB Perry, IM Nolte, JV van Vliet-Ostaptchouk, H Snieder, The LifeLines Cohort Study, T Esko, L Milani, R Mägi, A Metspalu, A Hamsten, PKE Magnusson, NL Pedersen, E Ingelsson, N Soranzo, MC Keller, NR Wray, ME Goddard, and PM Visscher. Genetic variance estimation with imputed variants finds negligible missing heritability for human height and body mass index. *Nat Gen*, 47:1114–1120, 2015.

[15] J Yang, B Benyamin, BP McEvoy, S Gordon, AK Henders, DR Nyholt, PA Madden, AC Heath, NG Martin, GW Montgomery, ME Goddard, and PM Visscher. Common SNPs explain a large proportion of the heritability for human height. *Nat Gen*, 42:565–569, 2010.

[16] J Yang, SH Lee, ME Goddard, and PM Visscher. GCTA: a tool for genome-wide complex trait analysis. *Am J Hum Genet*, 88:76–82, 2011.

[17] N Zaitlen and P Kraft. Heritability in the genome-wide association era. *Hum Gen*, 131:1655–1664, 2012.

[18] X Zhou and M Stephens. Genome-wide efficient mixed-model analysis for association studies. *Nat Gen*, 44:821–824, 2012.

[19] X Zhou and M Stephens. Efficient multivariate linear mixed model algorithms for genome-wide association studies. *Nat Methods*, 11:407–409, 2014.
